# Increasing cognitive load attenuates right arm swing in healthy human walking

**DOI:** 10.1098/rsos.160993

**Published:** 2017-01-25

**Authors:** Tim Killeen, Christopher S. Easthope, Linard Filli, Lilla Lőrincz, Miriam Schrafl-Altermatt, Peter Brugger, Michael Linnebank, Armin Curt, Björn Zörner, Marc Bolliger

**Affiliations:** 1 Spinal Cord Injury Center, University Hospital Balgrist, Forchstrasse 340, 8008 Zurich, Switzerland; 2 Department of Neurology, University Hospital Zurich, Frauenklinikstrasse 26, 8091 Zurich, Switzerland; 3 Department of Neurology, Helios-Klinik Hagen-Ambrock, Ambrocker Weg 60, 58091 Hagen, Germany

**Keywords:** arm swing, central pattern generator, cognitive control, dual-task, gender, motor control

## Abstract

Human arm swing looks and feels highly automated, yet it is increasingly apparent that higher centres, including the cortex, are involved in many aspects of locomotor control. The addition of a cognitive task increases arm swing asymmetry during walking, but the characteristics and mechanism of this asymmetry are unclear. We hypothesized that this effect is lateralized and a Stroop word-colour naming task—primarily involving left hemisphere structures—would reduce right arm swing only. We recorded gait in 83 healthy subjects aged 18–80 walking normally on a treadmill and while performing a congruent and incongruent Stroop task. The primary measure of arm swing asymmetry—an index based on both three-dimensional wrist trajectories in which positive values indicate proportionally smaller movements on the right—increased significantly under dual-task conditions in those aged 40–59 and further still in the over-60s, driven by reduced right arm flexion. Right arm swing attenuation appears to be the norm in humans performing a locomotor-cognitive dual-task, confirming a prominent role of the brain in locomotor behaviour. Women under 60 are surprisingly resistant to this effect, revealing unexpected gender differences atop the hierarchical chain of locomotor control.

## 1. Background

At all walking speeds, arm swing in human gait is driven at least partially by muscle activity [1–3]. While spinal central pattern generator (CPG) networks are implicated in arm swing generation and maintenance [3–5], recent evidence also supports a motor cortex contribution via the corticospinal tract [6]. Dual-task experiments, in which healthy participants walk while engaged in a secondary, cognitive task, have been observed to result in changes in the degree of arm swing amplitude relative to that of the contralateral arm—and thus the symmetry of the two movements [7–10].

The large majority of studies investigating arm swing symmetry examined the effect of walking conditions or disease states on an absolute arm swing symmetry index (ASI) calculated from a range of base parameters including sagittal shoulder angles or wrist trajectories, with most indices a variation on this calculation, in which *L* is the parameter of interest (e.g. sagittal shoulder angle) on the left and *R* that on the right:

$$\text{ABS}\;\left(\text{ASI}=\;\left(\frac{L-R}{\text{max}(L,R)}\right)\;\times 100\right),$$

which gives a scale of 0 (symmetrical) to 100 (maximally asymmetrical). Importantly, such metrics are agnostic to the direction of asymmetry and, as such, preclude the study of lateralized effects. When lateralized effects are sought, the absolute term is dropped, giving a scale of −100 (maximal right-dominant asymmetry) to 100 (maximum left-dominant asymmetry), with 0 representing perfect symmetry:

$$\text{ASI}=\left(\frac{L-R}{\text{max}(L,R)}\right)\;\times 100.$$

Intriguingly, studies of healthy walkers consistently describe a tendency for arm swing movements to be larger on the left [8,10–12]. Plate *et al*. recently investigated the effect of a serial subtraction task and a Stroop colour/word naming task during treadmill walking. While their main interest was absolute asymmetry changes, they noted that the Stroop task was associated with significantly more left-lateralized arm swing in healthy older walkers than the subtraction task [8]. We observed a similar tendency in a group of 12 young, healthy subjects walking on a treadmill while performing the Stroop task [10]. It remains, however, unclear whether a reduction in right arm swing amplitude or an increase on the left is responsible for these changes. The Stroop task, in which a participant must state the colour in which a colour-word is presented while suppressing its conflicting, written form, depends upon a number of brain structures critical for cognitive control, including prefrontal, cingulate and basal ganglia networks [11–14], some of which are also common to the control of gait [15–18] and arm swing [9,19]. As the Stroop task is predominantly a language exercise, its neural substrates are understood to be substantially lateralized, with left hemisphere structures activated more strongly than those on the right [11,20]. Cognitive-motor interference primarily in the left brain may therefore be responsible for the observed lateralized effect of the Stroop task on arm swing. While cognitive dual tasks, including the Stroop task, have been shown to have bilateral effects on lower limb kinematics [21], similar, unilateral effects either do not arise in the legs or are compensated for due to the need for maintaining symmetry for forward progression. The lumbar CPG may also be less susceptible to modulation of supraspinal influence than its cervical subsidiary involved in rhythmic arm movements [4,22,23]. In contrast to lower limb locomotor movements, arm swing may marginally increase the efficiency of gait but is not necessary for walking [2,24], and thus it is reasonable to assume that a lateralized response to interference from unilateral cognitive loading is more likely to manifest here. Confirmation of such measurably lateralized cognitive-motor interference in such a ubiquitous activity would be surprising and a potentially useful tool for the study of human motor control in health and disease.

If performing the Stroop task while walking is indeed associated with differential responses in the left and right arms, deliberate modulation of arm swing asymmetry in this way may provide information on the roles of cortical, subcortical and spinal networks in the control of locomotor arm movements. Attributes of the hitherto elusive human CPG may be studied by, for instance, assessing the phase-dependent characteristics of spinal reflexes [23] with and without the Stroop task or assessing the magnitude of the Stroop effect on arm swing in patients with spinal or brain lesions [9,10,25].

We aimed to build on the suggestive findings of Plate *et al*. [8] by confirming the presence of a lateralized effect of the Stroop task on arm swing in a large cohort of healthy individuals and establishing whether left or right swing amplitudes are affected. We use a modified version of the traditional Stroop task [26] using a pseudorandomized presentation frequency, designed to eliminate any entrainment of rhythmic gait parameters and encourage constant attention.

As ageing is associated with a deterioration in both motor and cognitive control, we expected the Stroop effect on arm swing to be greatest in older adults [27,28]. We were also interested in establishing whether this effect was dose-dependent by assessing arm swing during two Stroop tasks with different degrees of difficulty. We aimed to shed light on potential mechanisms for this effect by assessing whether cognitive loading exerts its effect during arm protraction, retraction or both. Finally, we examine whether factors such as gender confer susceptibility to any Stroop effect on ASI.

## 2. Material and methods

This two-centre study was approved by the Zurich cantonal ethics committee (KEK-2014-0004) and was carried out in accordance with the Declaration of Helsinki and Good Clinical Practice. Healthy volunteers aged 18–80 and blinded to the purpose of the study were recruited locally via flyers and a website and gave informed, written consent. Volunteers were paid 25 Swiss Francs (approx. 25.50 USD) per hour for their time and excluded if any abnormalities were present on medical screening, including colour-blindness. Participants were recruited into three age groups; 18–39, 40–59 and 60–80, with recruitment stopped once 20 males and 20 females were included in each group (21 females were ultimately included in the younger age group). All participants completed a laterality index (LI) questionnaire assessing indicators including preference for hand and foot use [29]. Included subjects underwent a 40 min acclimatization protocol including four, 45 s rehearsals of the dual-task conditions on the treadmill. The rest of the acclimatization period was spent practising treadmill walking tasks related to other studies.

Participants returned 1–7 days later for gait analysis. The timed 25 foot walk (T25FW) [30] and the 10 m walk test (10MWT) [31] were performed twice. Both tasks were performed simultaneously at maximum gait speed from a standing start in a hallway marked with both distances. The maximal overground speed (OG_max_) was calculated from the mean of the two T25FW attempts. Treadmill speed was set at 50% OG_max_ for all trials. Three-dimensional gait analysis (Vicon, UK) was conducted while walking on an instrumented treadmill (120 Hz, FDM-T, Zebris Medical GmbH, Germany) at one of two clinical gait laboratories. Data were recorded at 200 Hz using Nexus 1.8.5 (Vicon) motion capture software.

A reflective marker constellation was applied based on the Plug-in-Gait (Vicon) model [32] for the upper body and a modified Cleveland (Motion Analysis Corp., Santa Rosa, CA, USA) model for the pelvis and lower limbs [33].

Subjects walked barefoot without handrail support. Stable gait was recorded over 30–45 s. For the normal walking condition (NW), participants were asked to walk while fixating on a cross displayed on a 22^″^ LCD-monitor positioned 50 cm in front of the treadmill with its centre at eye height.

**Figure 1.**
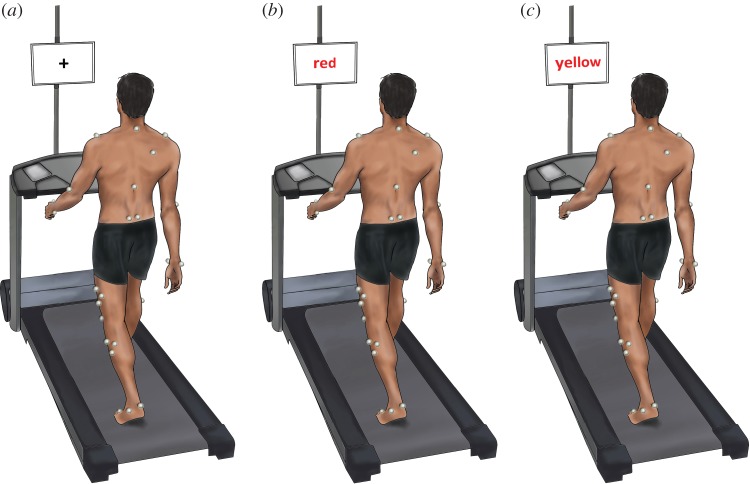
Experimental set-up. For the normal walking condition (*a*), subjects walked on an instrumented treadmill while fixating a black cross. They then performed two Stroop colour-naming task (see Material and methods) of differing difficulty. Image (*b*) shows the simpler task in which word and colour stimuli are congruent. In the more difficult, incongruent task (*c*) word and colour are discordant.

Cognitive distraction was achieved using a modified Stroop paradigm [26,34]. The presentations used are available as the electronic supplementary material (doi:10.5061/dryad.2kd0b). Two Stroop dual-task conditions were presented in the participant's self-declared native language and script (figure 1). In the congruent Stroop task (Stroop_easy_), the colouring of the text was consistent with that of the colour spelled out. In the incongruent task (Stroop_hard_), all stimuli consisted of spelled-out colours presented in a discordant colour. Incongruent colouring was randomly assigned with the additional requirement that no two sequential stimuli were of the same colour. Participants were told to read the word silently to themselves while verbally stating the colour and to continue immediately with the next stimulus should they fall behind with their responses. Trial order was fixed with NW followed by Stroop_easy_ and Stroop_hard_. The number of errors made during the Stroop task were recorded for each trial.

Data were reconstructed, labelled, filtered and modelled in Nexus. Lower body gait cycle events were set using treadmill force-plate data. Arm swing cycle was defined by maximal protraction and retraction of the modelled wrist joint centre (WJC)—a virtual point half-way between the two wrist markers—relative to the pelvis in the progression axis. ProCalc 1.1 (Vicon) was used to output spatio-temporal gait parameters.

The three-dimensional trajectory (in mean distance travelled per gait cycle) of the WJC was taken as the principal measure of arm swing behaviour [9]. This was used as the basis of a lateralized ASI (see formula in Background) [8,35] in which *L* is WJC trajectory on the left and *R* that on the right, giving a value between −100 and 100, with 0 representing perfectly symmetrical movements. Trials in which WJC trajectory was longer on the left yield a positive index, and vice versa.

ASI during normal treadmill walking varies considerably between individuals and this motivated our decision to exclude *a priori* from our analysis those with marked baseline asymmetry, with the rationale that any dual-task response may be masked by a ceiling effect if individuals exhibiting highly asymmetrical arm swing during NW were included. Based on observations in a pilot cohort [10], we therefore excluded subjects exhibiting significant asymmetry to either the left or the right during NW, defined as an ASI of less than −20 or more than 20.

Secondary outcome measures included the modelled sagittal shoulder angle cycle maxima and minima for the right and left upper limb to describe the contribution of sagittal extension and anteversion to any changes in ASI. These angles are relative to the trunk segment and are therefore theoretically unaffected by postural changes [32]. In the lower limb, we report step length, toe height at mid-swing and phase dispersion—a sensitive measure of interlimb coordination in which the timing of a given event is expressed as a percentage of the contralateral gait cycle [36,37]—for all limb pairs. To exclude anatomical differences in upper limb lengths as a confounding factor, left and right arm length was also calculated from the static calibration trial and an arm length symmetry index calculated.

Statistical analysis was performed using SPSS v. 23.0 (IBM Corp., Armonk, NY, USA) and graphs produced using Prism v. 7.1.0 (Graphpad Software, La Jolla, CA, USA). Gait parameters for each age group were analysed with a linear mixed model (LMM) in which condition (NW, Stroop_easy_, Stroop_hard_) was a repeated measure. Fixed effects comprised weight, height, gender, LI and walking speed. For significant effects, pairwise *t*-tests were performed across conditions with Bonferroni correction. Differences in demographic factors between groups were likewise assessed using an LMM without the condition factor. Correlations between ASI and Stroop performance were analysed using one-tailed Spearman's *ρ* after grouping all trials into three groups; no errors, one to five errors or more than five errors. An explorative, *post hoc* analysis of the effect of gender on ASI changes was performed using the same LMM approach. The relationship between laterality index and change in ASI between NW and Stroop_hard_ was assessed with Pearson's correlation coefficient. Statistical significance was set at *p* ≤ 0.05 for all tests.

## 3. Results

Of 145 volunteers, 24 were excluded at medical screening. Data were unusable for two subjects who stumbled during some trials. A further 36 exhibited marked baseline arm swing asymmetry and were excluded; 8 with right-dominant and 28 with left-dominant arm swing (electronic supplementary material, figure S1). Distribution of handedness and arm length symmetry was similar in both excluded groups and the main, included cohort. Mean baseline ASI for the whole cohort prior to the exclusion of these participants was 6.16 ± 1.75 (i.e. trajectories were approx. 6.6% longer on the left). More details of the full cohort including excluded participants are available in the electronic supplementary material, figures S1 and S2.

Eighty-three subjects were therefore included in the final analysis; 31 aged 18–39, 23 aged 40–59 and 29 aged 60–80. Group characteristics are summarized in table 1. Gender distribution was similar across age groups. Performance in walking tests declined with age, with longer times in both the T25FW (*p* = 0.008) and 10MWT (*p* = 0.013) in the 60–80 age group. As treadmill speed was determined by the 25FWT result, median walking speed was 4.3, 4.0 and 3.7 km h^−1^ in the younger, middle-aged and older groups, respectively (younger and older significantly different; *p* = 0.005). Discrepancies in arm length (left versus right) were negligible in all groups.

**Table 1.** Subject characteristics.

age group	*n*	age (years)	per cent female	weight (kg)	height (cm)	per cent right-handers	walking speed (km h^−1^; median ± IQR)	25FWT (s)	10MWT (s)	6MWT (m)	arm length asymmetry index (L dominance is positive)
18–39	31	28.6 ± 4.9	51.6	71.3 ± 15.5	173 ± 9	93.5	4.3 ± 0.9	3.28 ± 0.44	4.30 ± 0.61	726 ± 80	−0.58 ± 2.18
40–59	23	47.5 ± 6.1	52.0	73.5 ± 13.0	172 ± 8	96.0	4.0 ± 0.6	3.50 ± 0.47	4.62 ± 0.63	701 ± 83	−0.66 ± 1.89
60–80	29	67.8 ± 4.5	62.1	65.5 ± 10.0	168 ± 9	96.6	3.7 ± 0.6	3.76 ± 0.60	4.91 ± 0.82	671 ± 100	−0.26 ± 2.35

**Figure 2.**
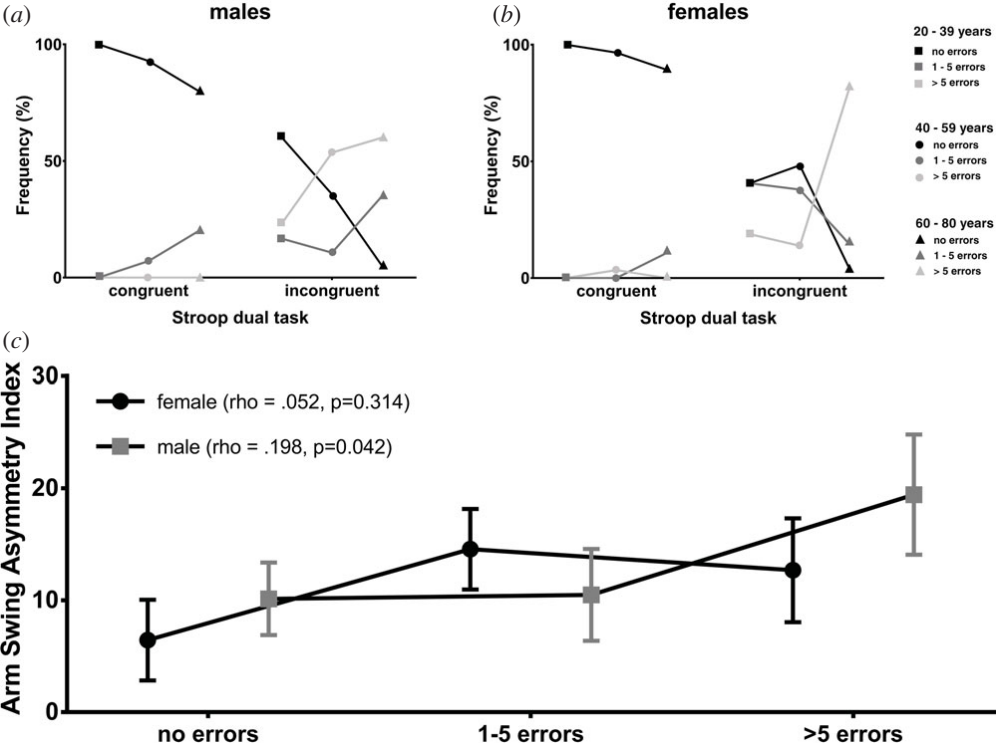
Performance in congruent and incongruent Stroop tasks; (*a*) males, (*b*) females. Relative frequency of Stroop trial error rate trichotomized into no errors, one to five errors or more than five errors. (*c*) Correlation (Spearman's *ρ*) between arm swing asymmetry index and error rate during the incongruent Stroop task.

Performance in both the congruent and incongruent Stroop tasks became worse with age (figure 2*a*,*b*). Errors were rare in the congruent task, with no 20–39 year olds making more than five mistakes. Males made more mistakes than their female counterparts in the congruent task. In the two younger age groups, men were more likely to make more than five errors during the incongruent task than women, but women's performance deteriorated significantly over 60 years of age during this task, in which 89.3% of older females and 80% of older males made more than five errors.

**Figure 3.**
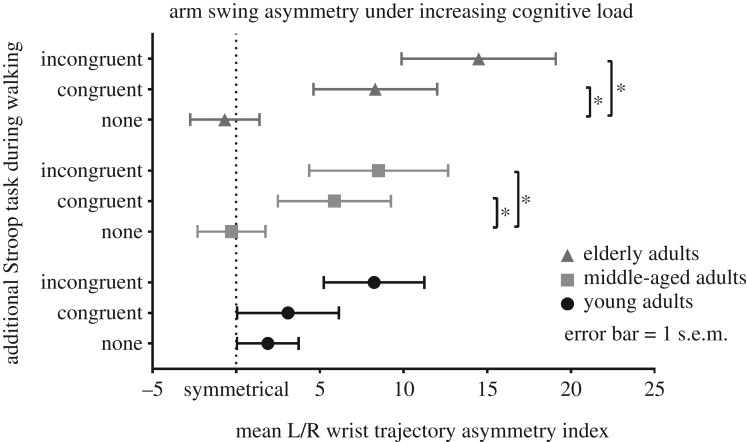
Arm swing asymmetry under increasing cognitive load. Wrist trajectory asymmetry index is calculated using the left and right three-dimensional wrist centre trajectories, with left dominance resulting in a positive value and vice versa. ASI is given as the mean value per gait cycle over a trial of 45 s (approx. 42 gait cycles at 4 km h^−1^). s.e.m., Standard error of the mean. Statistical significance was determined using a linear mixed model with *post hoc t*-tests. The *p*-values are corrected for multiple pairwise within-age group comparisons using the Bonferroni method.

Overall, ASI increased under cognitive load, from (mean ± s.e.m.) 0.37 ± 1.13 to 5.69 ± 1.94 in Stroop_easy_ and again to 10.49 ± 2.27 in Stroop_hard_. This represents left WJC trajectories, respectively, 0.4%, 6.0% and 11.7% larger than those on the right. All groups demonstrated the same tendency, with mean ASI increasing from 1.89 ± 1.83 to 3.10 ± 3.03 and 8.24 ± 3.01 in the 18–39 age group, −0.28 ± 2.03 to 5.87 ± 3.38 and 9.19 ± 3.31 in the 40–59 age group and −0.68 ± 2.07 to 8.31 ± 3.70 and 15.16 ± 3.80 in the 60–80 age group (figure 3). These increases in ASI between normal walking (NW) and Stroop_easy_ were significant for middle-aged (*p* = 0.048) and older adults (*p* = 0.009), as were those between NW and Stroop_hard_ in the middle-aged (*p* = 0.009) and older (*p* < 0.000) groups. Overall, ASI did not correlate with Stroop performance (*ρ* = 0.114, *p* = 0.071) but there was a moderate correlation in males (*ρ* = 0.198, *p* = 0.042; figure 2*c*).

**Figure 4.**
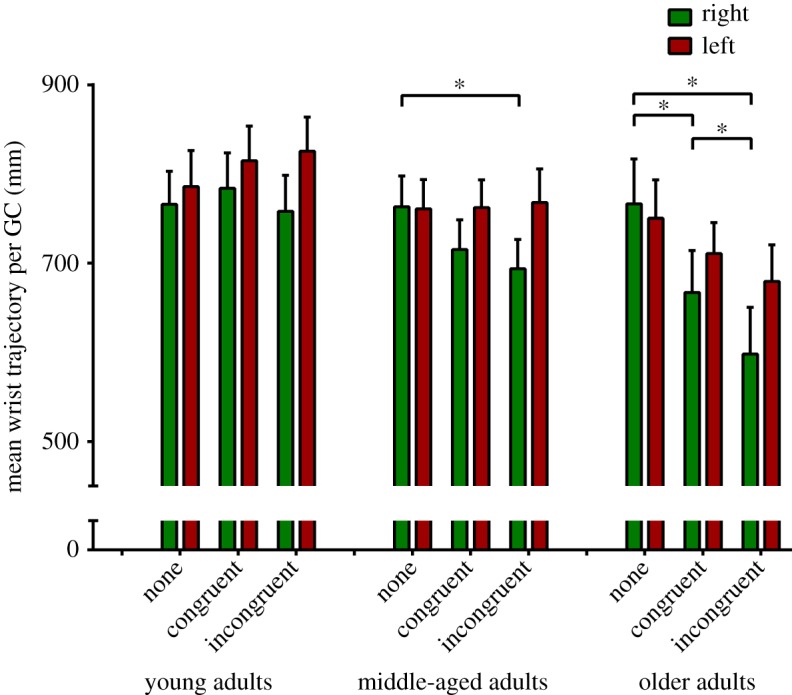
Absolute wrist trajectory length. Three-dimensional wrist joint centre trajectories for younger, middle-aged and older adults during normal walking and during a congruent and an incongruent Stroop dual-task. GC, gait cycle. Error bars indicate 1 s.e.m. Statistical significance was determined using a linear mixed model with *post hoc t*-tests. The *p*-values are corrected for multiple pairwise within-age group comparisons using the Bonferroni method.

Changes in asymmetry were driven by reductions in right wrist joint centre (WJC) trajectory relative to those on the left (figure 4). This reduction in mean right arm swing relative to baseline was significant in the older age group, in which right trajectory length decreased by 13.1% (*p* = 0.049) and 22.1% (*p* < 0.000) during Stroop_easy_ and Stroop_hard_ conditions, respectively, with the reduction between the dual-tasks also significant (*p* = 0.035).

**Figure 5.**
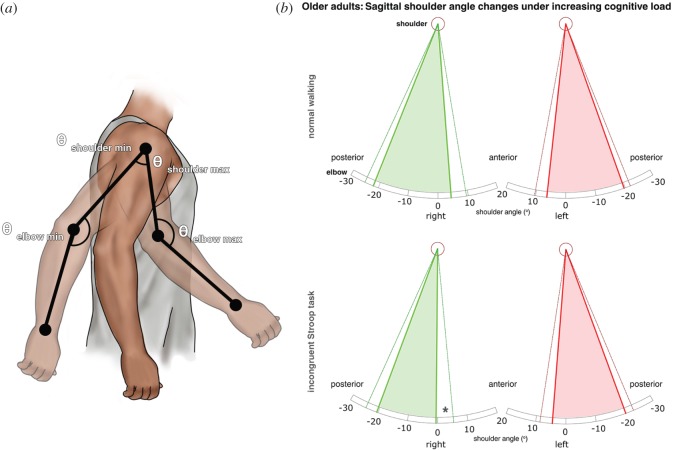
(*a*) Sagittal gait cycle mean joint angle maxima and minima based on the approach used by Roggendorf *et al*. [25]. (*b*) Sagittal shoulder angle changes during normal walking and under increased cognitive load (incongruent Stroop task) in older adults walking on a treadmill. Diagrams represent the right (green) and left (red) mean sagittal shoulder angle maxima and minima (thick lines) per gait cycle with associated single standard deviations (thin dark lines). A significant decrease in shoulder flexion in the incongruent Stroop task is indicated by asterisk (*). Elbow flexion was also reduced under increased cognitive load, with preserved extension (not shown; see Results section).

Sagittal shoulder and elbow angles were analysed to determine whether arm swing amplitude changes were occurring during arm protraction, retraction or both (figure 5*a*). In older adults, maximal shoulder anteversion decreased (mean ± s.d.; NW: 4.35 ± 10.07°, Stroop_easy_: 1.21 ± 10.35°, Stroop_hard_: −0.54 ± 11.62°; *p* ≤ 0.032) under both conditions, while right maximal elbow flexion also reduced between the NW and Stroop_hard_ conditions (figure 5*b*; NW: 53.69 ± 11.49°, Stroop_hard_: 48.34 ± 8.08°; *p* = 0.012). A significant effect was seen neither during flexion on the left, nor in extension bilaterally. In the two younger age groups, trends towards decreases in right shoulder and elbow flexion did not reach significance (data not shown). In the younger age group in whom a non-significant increase in left WJC trajectory was seen (figure 4), this was manifested as a trend towards increases in both extension and flexion maxima.

**Figure 6.**
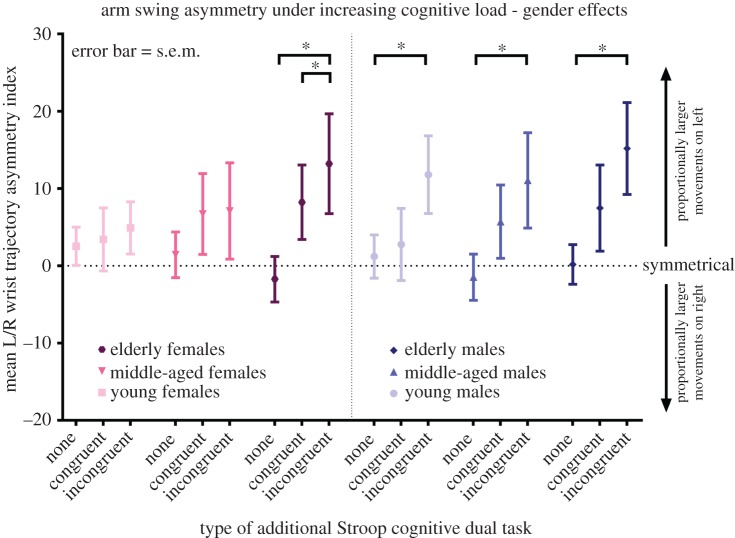
Arm swing asymmetry under increasing cognitive load—gender effects. Wrist trajectory asymmetry index is calculated using the left and right three-dimensional wrist centre trajectories, with left dominance resulting in a positive value and vice versa. ASI is given as the mean value per gait cycle over a trial of 45 s (approx. 42 gait cycles at 4 km h^−1^), s.e.m.; standard error of the mean. Statistical significance was determined using a linear mixed model with *post hoc t*-tests. The *p*-values are corrected for pairwise within-group comparisons using the Bonferroni method.

While, in the older age group, both genders responded similarly to the addition of the Stroop task with increases in ASI (males; NW: 0.17 ± 2.57, Stroop_easy_: 7.47 ± 5.58; *p* = ns, Stroop_hard_: 15.19 ± 5.95; *p* = 0.09, females; NW: −1.73 ± 2.94, Stroop_easy_: 8.23 ± 4.82; *p* = 0.021, Stroop_hard_: 13.21 ± 6.46; *p* = 0.003), gender differences were observed in the two younger age groups (figure 6). Males aged 18–39 showed a significant increase in ASI relative to NW during Stroop_hard_ (1.20 ± 2.80 to 11.79 ± 5.03; *p* = 0.047). Similarly, males aged 40–59 exhibited significant leftward shifts in ASI from −1.47 ± 2.98 to 5.71 ± 4.75 in Stroop_easy_ (*p* = 0.044) and 11.05 ± 6.17 in Stroop_hard_ (*p* = 0.010). Females in both younger groups showed no significant changes in ASI.

The cohort included only four left-handers, including two educated as right-handers. Mean (±s.d.) laterality index (−13 = fully left lateralized, 13 = fully right) was 8.5 ± 4.0. There was no correlation between laterality index (neither the full index nor its motor subcomponents) and changes in ASI under increased cognitive load.

Changes in lower body spatio-temporal parameters are summarized in the electronic supplementary material, table S1 and figure S3. Gait in older adults during Stroop_hard_ was characterized by increased stride-to-stride step length variability and small but significant reductions in foot clearance bilaterally. Stride time and stride time variability was unaffected by condition or age group. Measures of interlimb temporal coordination remained unchanged across conditions (electronic supplementary material, figure S4).

## 4. Discussion

As expected, our cohort broadly replicates findings relating to the effect of the Stroop and other cognitive dual-tasks on lower body kinematics [17,21,38], with healthy older subjects exhibiting a marked increase in step length variability and decreased foot clearance under increased cognitive load (electronic supplementary material, table S1, figures S3 and S4). We contribute the novel finding that increasing cognitive loading in a language task during treadmill walking results in a corresponding, dose-dependent increase in left-lateralized arm swing. This effect is age- and sex-dependent, with men of all ages susceptible to enhanced asymmetry under cognitive load, while women under the age of 60 are resistant. The asymmetry shift is driven by an attenuation of right arm swing amplitudes and, at least in older adults, by reduced flexion at the right shoulder and elbow during limb protraction.

Recent work reported the surprising finding that ASI is modulated through both the Stroop task and a serial sevens counting paradigm in 60 healthy adults [8]. The authors noted a left-dominant arm swing asymmetry, significantly stronger in the Stroop task than during serial subtraction, and speculated that the additional left hemispheric language processing of the former underlay this difference. Whether reductions in right or increases in left swing magnitude were responsible for this shift was not stated. The data presented here clearly confirm that such asymmetry, provoked by the Stroop task, is indeed directional and is driven by a significant decrease in arm swing amplitude on the right. We also show that the degree of right arm swing attenuation is modulated by task difficulty, age and gender, with those aged 60 and above consistently showing the largest shifts in ASI in Stroop_hard_. Supraspinal processing thus indeed appears to result in a dose-dependent paucity of right arm swing during walking.

### 4.1. Suppression of arm flexors

The Stroop task may result in reduced left supraspinal drive acting upon the right cervical part of the locomotor pattern generator underlying arm swing, with candidate sites of upstream interference the left frontal regions and basal ganglia circuits common to both the control of locomotor behaviour and cognitive control [11–14,19,20,39,40]. It has been proposed that arm movements are regulated by task-dependent neuronal coupling [3,41,42], through which the rhythmic activation of cervical CPG networks is facilitated during locomotion and inhibited when goal-directed upper limb control is desired [22,43]. Such unilateral gating allows locomotor movements to continue in one arm, while the other is engaged in a skilled motor task (e.g. while gesticulating or manipulating an object). The predominantly verbal Stroop dual-task may thus lead, through predominant activation of left cortical and basal ganglia structures [14,44], to suppression of rhythmic locomotor activity in the right arm via a non-specific decrease in supraspinal drive.

Alternatively, and in keeping with some evidence that Stroop performance causes interference in both hemispheres [13,20], dual-task processing may lead to cortically mediated, decreased drive to spinal centres, the effects of which only manifest in the dominant arm. This interpretation is supported by the existence of an apparent tendency towards asymmetrical, left-dominant arm swing during normal treadmill walking (i.e. without a dual-task) in this and previous reports [8,10,35,45]. This puzzling baseline asymmetry may result from the attentional demands associated with walking in unhabituated conditions [46,47], although our participants underwent lengthy treadmill acclimatization. There was an insufficient number of left-handers in our sample to test this hypothesis, but others have demonstrated that directional arm swing asymmetry is not related to handedness during normal treadmill walking [8,35] and there was no correlation between ASI changes and LI in our overwhelmingly right-handed cohort. Why dominant arm swing amplitude should be more sensitive to decreased bihemispheric supraspinal drive is not clear. Further research, considering the full complexity of human handedness and its interplay with cognition (e.g. left-handedness with preserved versus reversed hemispheric language specialization), would be of value.

Interestingly, asymmetry in older adults—the group most affected by the Stroop task—was driven by a reduction in shoulder and elbow flexion and may be reflective of a unilateral unmasking of an extensor-dominant CPG [48] as cortical drive is reallocated away from locomotion. In the legs, a dissociation exists between more centrally modulated flexor activity and extensors under the control of more autonomous, proprioceptive pathways [49]. An analogous situation may exist in the arms, where differential corticospinal projection to biceps and triceps mirrors that in the tibialis anterior and soleus muscles [50,51]. More convincingly, this flexor/extensor dissociation may arise from reduced, corticospinally modulated biceps activity in the context of unimpaired extension under the control of the reticulospinal tract.

### 4.2. Facilitation of arm extensors

An alternative explanation assumes that, rather than decreasing supraspinal drive, engaging in the Stroop task augments cortical inputs to the contralateral upper limb compared to NW. While both proximal flexors and extensors are active during arm swing, it is believed that forward swing is more passive than retraction and that eccentric extensor activation is used to arrest arm protraction prior to swing reversal [3,52]. Inhibitory transcranial magnetic stimulation reduces electromyographical (EMG) activity in the posterior deltoid during arm swing [6], so it follows that facilitation would result in reduced shoulder anteversion under cognitive load. Ascertaining the effect of the Stroop task on upper limb EMG would be illuminating.

Arm swing asymmetry in Parkinson's disease (PD) patients is, in contrast to the reduction in flexion seen here, characterized by deficits in shoulder *extension* [9,25]. Asymmetry in PD may therefore be due to an entirely different, pathological mechanism such as rigidity. Establishing the relative contributions of flexion and extension to ASI in PD may prove an improved diagnostic criterion, as, while (absolute) ASI appears to be a sensitive indicator of early PD [9], its specificity is hampered by high variability in healthy controls.

### 4.3. Gender

A gender sub-analysis showed that the effect of ageing on ASI shift was specific to females (figure 3). This unexpected and pronounced resistance to right arm swing attenuation in pre-menopausal women may be due to oestrogen-mediated plasticity and attendant redundancy in the prefrontal cortex (PFC), where oestrogen receptors are plentiful [53] and oestradiol increases dendritic spine density in primates [54]. In women, there is an oestrogen-related enhancement of cognitive control and inhibition of inappropriate responses [55,56], while susceptibility to the Stroop task is ameliorated by oestrogen treatment after the menopause [57]. The left PFC—widely implicated in cognitive control [58] and activated during active stepping [18], treadmill walking [59] and the Stroop task [11,60]—is thus a strong candidate for the site of the cognitive-motor interference underlying the observed ASI shifts in men and older women. Targeting of the left PFC with oestrogen therapy or transcranial magnetic stimulation [61] may improve motor control in elderly fallers and patients with gait instability.

It is noteworthy that the increase in ASI observed in young males (figure 5) is, in contrast to those in the older age groups, driven by an absolute increase in left arm swing, with a relatively smaller reduction on the right (figure 3). While females in this group slightly increase their arm swing bilaterally, males failed to do so on the right, accounting for their increased, left-dominant asymmetry.

### 4.4. Interlimb coordination

Interlimb coordination was highly preserved irrespective of age or gender and despite the significant changes in the spatial characteristics of arm movements under increased cognitive load. That the timing of key temporal elements of arm swing is not affected by cognitive load is in keeping with a predominantly spinal source of interlimb coordination along propriospinal connections between the cervical and lumbar CPG elements [22,41,62,63].

### 4.5. Methodological considerations

The dual-task methodology presented here differs slightly from that of previous researchers [8] and may account for the strength of the results. Presenting rhythmic stimuli risks stimulus-motor entrainment [64,65], so we chose to present Stroop cues at irregular intervals around a mean frequency of 1 Hz (range 600–1400 ms). This had the additional, beneficial effect of making the Stroop task harder and requires constant, rather than intermittent, attention as subjects could not anticipate stimulus duration.

Walking speed was set as 50% of OG_max_, approximating preferred speed, at which arm swing is well established and in phase with the legs. This resulted in different median speeds in the three age groups. However, while arm swing amplitude varies as a function of gait speed, ASI does not [8]. In practice, the difference in median speeds was small and mean amplitudes were similar during NW in all three groups (figure 2).

### 4.6. Excluded subjects

Determining an appropriate cut-off value for exclusion in experiments measuring ASI is difficult for a number of reasons, especially as a degree of left-dominant asymmetry appears to be physiological during normal treadmill walking [8,10,35,45]. The approaches taken by different research groups to calculate ASI are diverse, with various two-dimensional sagittal metrics most commonly used in conjunction with two competing calculations; the ASI and asymmetry angle [66,67]. Furthermore, an absolute, non-directional ASI is used by many authors whose hypotheses do not investigate laterality. Unfortunately, the only study using comparable, three-dimensional trajectories to describe asymmetry [9] is of limited usefulness in informing cut-off values in our cohort as it featured overground walking and used an absolute asymmetry angle. We therefore referred to data from a previous study in our laboratory [10], in which three of 12 healthy control subjects who exhibited normal walking ASI values of +20 or more showed evidence of a ceiling effect under cognitive loading, to set inclusion baseline ASI values of −20 to +20.

No consistent dual-task effect was observed amongst the 28 subjects excluded for baseline ASI values over 20 (electronic supplementary material, figure S1). Individual responses were highly variable (electronic supplementary material, figure S2). Only 36% of individuals in this group showed an ASI shift between NW and Stroop_hard_ of more than 10, compared with 46% of those in the included group (electronic supplementary material, figure S2). This fact, and the observation that variance was 13% smaller in the group with ASI over 20 compared with the included group, suggest that a ceiling effect indeed contributed to the findings observed in this group. The eight individuals with strongly right-dominant asymmetry during NW (less than −20) showed no common response to cognitive load (electronic supplementary material, figures S1 and S2).

Some healthy individuals may exhibit marked baseline asymmetry due to acquired atypical gait patterns [68] and the possibility exists that some baseline asymmetry may have been a subclinical manifestation of neurological or musculoskeletal disease in our participants. Both causes of asymmetry may result in different responses to cognitive load.

### 4.7. Outlook: right hemisphere dual-tasks

It would be interesting to apply this approach with a task subsisting on primarily right hemisphere structures, with the expectation that reciprocal effects on left arm swing may be observed. To this end, our group has performed pilot experiments with an increasingly difficult bisecting lines task (Landmark task) [69] in individuals known to be susceptible to the ASI Stroop effect, with negative results. However, this task does not recruit right prefrontal networks [70] so future experiments with an appropriate paradigm may be more successful.

## 5. Conclusion

Reduction in right arm swing appears to be the norm in humans performing a motor-cognitive dual-task, confirming a prominent role of the brain in arm swing behaviour. In older adults, asymmetry is characterized by reduced arm protraction, suggesting that upper limb flexors are under more direct supraspinal control and susceptible to interference. Alternatively, the Stroop task may enhance cortical drive to the right arm extensors, braking passive shoulder anteversion through enhanced eccentric extensor contraction. Overcoming this interference appears to be a trait unique to younger females and implies significant gender differences at the top of the hierarchical chain of locomotor control. Applying this paradigm to patients with PD, subcortical stroke and spinal cord injury may permit further insights into the control of arm movements in human locomotion.

## Supplementary Material

Supplementary Figure 1

## Supplementary Material

Supplementary Figure 2

## Supplementary Material

Supplementary Figure 3

## Supplementary Material

Supplementary Figure 4

## Supplementary Material

Supplementary Table 1

## Data accessibility

Supporting data and the Stroop stimuli used in this experiment are available at the Dryad Digital Repository: http://dx.doi.org/10.5061/dryad.2kd0b [71].

## References

[1] Goudriaan M, Jonkers I, van Dieen JH, Bruijn SM 2014 Arm swing in human walking: what is their drive? Gait Posture 40, 321–326. (doi:10.1016/j.gaitpost.2014.04.204) 24865637 10.1016/j.gaitpost.2014.04.204

[2] Collins SH, Adamczyk PG, Kuo AD 2009 Dynamic arm swinging in human walking. Proc. R. Soc. B 276, 3679–3688. (doi:10.1098/rspb.2009.0664) 10.1098/rspb.2009.0664 PMC2817299 19640879

[3] Kuhtz-Buschbeck JP, Jing B 2012 Activity of upper limb muscles during human walking. J. Electromyogr. Kinesiol. 22, 199–206. (doi:10.1016/j.jelekin.2011.08.014) 21945656 10.1016/j.jelekin.2011.08.014

[4] Solopova IA, Selionov VA, Zhvansky DS, Gurfinkel VS, Ivanenko Y 2016 Human cervical spinal cord circuitry activated by tonic input can generate rhythmic arm movements. J. Neurophysiol. 115, 1018–1030. (doi:10.1152/jn.00897.2015) 26683072 10.1152/jn.00897.2015

[5] Dietz V 2002 Do human bipeds use quadrupedal coordination? Trends Neurosci. 25, 462–467. (doi:10.1016/S0166-2236(02)02229-4) 12183207 10.1016/s0166-2236(02)02229-4

[6] Barthelemy D, Nielsen JB 2010 Corticospinal contribution to arm muscle activity during human walking. J. Physiol. 588, 967–979. (doi:10.1113/jphysiol.2009.185520) 20123782 10.1113/jphysiol.2009.185520 PMC2849962

[7] Mirelman A, Bernad-Elazari H, Nobel T, Thaler A, Peruzzi A, Plotnik M, Giladi N, Hausdorff JM 2015 Effects of aging on arm swing during gait: the role of gait speed and dual tasking. PLoS ONE 10, e0136043 (doi:10.1371/journal.pone.0136043) 26305896 10.1371/journal.pone.0136043 PMC4549059

[8] Plate A, Sedunko D, Pelykh O, Schlick C, Ilmberger JR, Bötzel K 2015 Normative data for arm swing asymmetry: how (a)symmetrical are we? Gait Posture 41, 13–18. (doi:10.1016/j.gaitpost.2014.07.011) 25442669 10.1016/j.gaitpost.2014.07.011

[9] Lewek MD, Poole R, Johnson J, Halawa O, Huang X 2010 Arm swing magnitude and asymmetry during gait in the early stages of Parkinson's disease. Gait Posture 31, 256–260. (doi:10.1016/j.gaitpost.2009.10.013) 19945285 10.1016/j.gaitpost.2009.10.013 PMC2818433

[10] Killeen T, Easthope CS, Filli L, Linnebank M, Curt A, Bolliger M, Zörner B 2016 Modulating arm swing symmetry with cognitive load: a window on rhythmic spinal locomotor networks in humans? J. Neurotrauma (Epub ahead of print) (doi:10.1089/neu.2016.4554) 10.1089/neu.2016.4554 27574966

[11] Milham MP, Erickson KI, Banich MT, Kramer AF, Webb A, Wszalek T, Cohen NJ 2002 Attentional control in the aging brain: insights from an fMRI study of the Stroop task. Brain Cogn. 49, 277–296. (doi:10.1006/brcg.2001.1501) 12139955 10.1006/brcg.2001.1501

[12] Mohtasib RS, Lumley G, Goodwin JA, Emsley HCA, Sluming V, Parkes LM 2012 Calibrated fMRI during a cognitive Stroop task reveals reduced metabolic response with increasing age. Neuroimage 59, 1143–1151. (doi:10.1016/j.neuroimage.2011.07.092) 21843646 10.1016/j.neuroimage.2011.07.092

[13] Leung HC, Skudlarski P, Gatenby JC, Peterson BS, Gore JC 2000 An event-related functional MRI study of the Stroop color word interference task. Cereb. Cortex 10, 552–560. (doi:10859133) 10859133 10.1093/cercor/10.6.552

[14] Stafford T, Gurney K 2005 The basal ganglia as the selection mechanism in a cognitive task. In Modelling natural action selection (eds JJ Bryson, TJ Prescott, A Seth), pp. 77–83. Edinburgh, UK: AISB Press.

[15] Seidler RD, Bernard JA, Burutolu TB, Fling BW, Gordon MT, Gwin JT, Kwak Y, Lipps DB 2011 Motor control and aging: links to age-related brain structural, functional and biomechanical effects. Neurosci. Biobehav. Rev. 34, 721–733. (doi:10.1016/j.neubiorev.2009.10.005.Motor) 10.1016/j.neubiorev.2009.10.005 PMC2838968 19850077

[16] Dietz V 1992 Human neuronal control of automatic functional movements: interaction between central programs and afferent input. Physiol. Rev. 72, 33–69. 1731372 10.1152/physrev.1992.72.1.33

[17] Hausdorff JM, Yogev G, Springer S, Simon ES, Giladi N 2005 Walking is more like catching than tapping: gait in the elderly as a complex cognitive task. Exp. Brain Res. 164, 541–548. (doi:10.1007/s00221-005-2280-3) 15864565 10.1007/s00221-005-2280-3

[18] Jaeger L, Marchal-Crespo L, Wolf P, Riener R, Michels L, Kollias S 2014 Brain activation associated with active and passive lower limb stepping. Front. Hum. Neurosci. 8, 828 (doi:10.3389/fnhum.2014.00828) 25389396 10.3389/fnhum.2014.00828 PMC4211402

[19] Crenna P, Carpinella I, Lopiano L, Marzegan A, Rabuffetti M, Rizzone M, Lanotte M, Ferrarin M 2008 Influence of basal ganglia on upper limb locomotor synergies. Evidence from deep brain stimulation and L-DOPA treatment in Parkinson's disease. Brain 131, 3410–3420. (doi:10.1093/brain/awn272) 18952669 10.1093/brain/awn272

[20] Belanger HG, Cimino CR 2002 The lateralized Stroop: a meta-analysis and its implications for models of semantic processing. Brain Lang. 83, 384–402. (doi:10.1016/S0093-934X(02)00508-4) 12468395 10.1016/s0093-934x(02)00508-4

[21] Al-Yahya E, Dawes H, Smith L, Dennis A, Howells K, Cockburn J 2011 Cognitive motor interference while walking: a systematic review and meta-analysis. Neurosci. Biobehav. Rev. 35, 715–728. (doi:10.1016/j.neubiorev.2010.08.008) 20833198 10.1016/j.neubiorev.2010.08.008

[22] Falgairolle M, de Seze M, Juvin L, Morin D, Cazalets JR 2006 Coordinated network functioning in the spinal cord: an evolutionary perspective. J. Physiol. Paris 100, 304–316. (doi:10.1016/j.jphysparis.2007.05.003) 17658245 10.1016/j.jphysparis.2007.05.003

[23] Zehr EP, Carroll TJ, Chua R, Collins DF, Frigon A, Haridas C, Hundza SR, Thompson AK 2004 Possible contributions of CPG activity to the control of rhythmic human arm movement. Can. J. Physiol. Pharmacol. 82, 556–568. (doi:10.1139/y04-056) 15523513 10.1139/y04-056

[24] Umberger BR 2008 Effects of suppressing arm swing on kinematics, kinetics, and energetics of human walking. J. Biomech. 41, 2575–2580. (doi:10.1016/j.jbiomech.2008.05.024) 18621376 10.1016/j.jbiomech.2008.05.024

[25] Roggendorf J, Chen S, Baudrexel S, van de Loo S, Seifried C, Hilker R 2012 Arm swing asymmetry in Parkinson's disease measured with ultrasound based motion analysis during treadmill gait. Gait Posture 35, 116–120. (doi:10.1016/j.gaitpost.2011.08.020) 21962405 10.1016/j.gaitpost.2011.08.020

[26] Stroop J 1935 Studies of interference in serial verbal reactions. J. Exp. Psychol. 121, 15–23. (doi:10.1037/0096-3445.121.1.15)

[27] Cohn NB, Dustman RE, Bradford DC 1984 Age-related decrements in Stroop color test performance. J. Clin. Psychol. 40, 1244–1250. (doi:10.1002/1097-4679(198409)40:5<1244::AID-JCLP2270400521>3.0.CO;2-D) 6490922 10.1002/1097-4679(198409)40:5<1244::aid-jclp2270400521>3.0.co;2-d

[28] West R, Alain C 2000 Age-related decline in inhibitory control contributes to the increased Stroop effect observed in older adults. Psychophysiology 37, 179–189. (doi:10.1017/S0048577200981460) 10731768

[29] Coren S, Porac C, Duncan P 1979 A behaviorally validated self-report inventory to assess four types of lateral preference. J. Clin. Neuropsychol. 1, 55–64. (doi:10.1080/01688637908401098)

[30] Bohannon R 1997 Comfortable and maximum walking speed of adults aged 20–79 years: reference values and determinants. Age Ageing 26, 15–19. (doi:10.1093/ageing/26.1.15) 10.1093/ageing/26.1.15 9143432

[31] Rossier P, Wade DT 2001 Validity and reliability comparison of 4 mobility measures in patients presenting with neurologic impairment. Arch. Phys. Med. Rehabil. 82, 9–13. (doi:10.1053/apmr.2001.9396) 11239279 10.1053/apmr.2001.9396

[32] Vicon Motion Systems. 2010 Plug-in Gait Product Guide.

[33] Svoboda B, Kranzl A 2012 A study of the reproducibility of the marker application of the Cleveland Clinic Marker Set including the Plug-In Gait Upper Body Model in clinical gait analysis. Gait Posture 36, S62–S63. (doi:10.1016/j.gaitpost.2011.10.286)

[34] Macleod CM 1991 Half a century of research on the Stroop effect: an integrative review. Psychol. Bull. 109, 163–203. (doi:10.1037/0033-2909.109.2.163) 2034749 10.1037/0033-2909.109.2.163

[35] Kuhtz-Buschbeck JP, Brockmann K, Gilster R, Koch A, Stolze H 2008 Asymmetry of arm-swing not related to handedness. Gait Posture 27, 447–454. (doi:10.1016/j.gaitpost.2007.05.011) 17616462 10.1016/j.gaitpost.2007.05.011

[36] Kloos AD, Fisher LC, Detloff MR, Hassenzahl DL, Basso DM 2005 Stepwise motor and all-or-none sensory recovery is associated with nonlinear sparing after incremental spinal cord injury in rats. Exp. Neurol. 191, 251–265. (doi:10.1016/j.expneurol.2004.09.016) 15649480 10.1016/j.expneurol.2004.09.016

[37] Filli L, Zörner B, Weinmann O, Schwab ME 2011 Motor deficits and recovery in rats with unilateral spinal cord hemisection mimic the Brown-Séquard syndrome. Brain 134, 2261–2273. (doi:10.1093/brain/awr167) 21752788 10.1093/brain/awr167

[38] Hamacher D, Hamacher D, Schega L 2014 Towards the importance of minimum toe clearance in level ground walking in a healthy elderly population. Gait Posture 40, 727–729. (doi:10.1016/j.gaitpost.2014.07.016) 25128155 10.1016/j.gaitpost.2014.07.016

[39] Meester D, Al-Yahya E, Dawes H, Martin-Fagg P, Piñon C 2014 Associations between prefrontal cortex activation and H-reflex modulation during dual task gait. Front. Hum. Neurosci. 8, 78 (doi:10.3389/fnhum.2014.00078) 24600375 10.3389/fnhum.2014.00078 PMC3926984

[40] Jahn K, Deutschländer A, Stephan T, Strupp M, Wiesmann M, Brandt T 2004 Brain activation patterns during imagined stance and locomotion in functional magnetic resonance imaging. Neuroimage 22, 1722–1731. (doi:10.1016/j.neuroimage.2004.05.017) 15275928 10.1016/j.neuroimage.2004.05.017

[41] Dietz V, Fouad K, Bastiaanse CM 2001 Neuronal coordination of arm and leg movements during human locomotion. Eur. J. Neurosci. 14, 1906–1914. (doi:10.1046/j.0953-816x.2001.01813.x) 11860485 10.1046/j.0953-816x.2001.01813.x

[42] Haridas C, Zehr EP 2003 Coordinated interlimb compensatory responses to electrical stimulation of cutaneous nerves in the hand and foot during walking. J. Neurophysiol. 90, 2850–2861. (doi:10.1152/jn.00531.2003) 12853441 10.1152/jn.00531.2003

[43] Lundberg A 1999 Descending control of forelimb movements in the cat. Brain Res. Bull. 50, 323–324. (doi:10.1016/S0361-9230(99)00151-3) 10643418 10.1016/s0361-9230(99)00151-3

[44] Perret E 1974 The left frontal lobe of man and the suppression of habitual responses in verbal categorical behaviour. Neuropsychologia 12, 323–330. (doi:10.1016/0028-3932(74)90047-5) 4421777 10.1016/0028-3932(74)90047-5

[45] Riley TL, Ray WF, Massey EW 1977 Gait mechanisms: asymmetry of arm motion in normal subjects. Mil. Med. 142, 467–468. 407495

[46] Regnaux JP, Roberston J, Smail D, Daniel O, Bussel B 2006 Human treadmill walking needs attention. J. Neuroeng. Rehabil. 3, 19 (doi:10.1186/1743-0003-3-19) 16923186 10.1186/1743-0003-3-19 PMC1564141

[47] Clark DJ, Christou EA, Ring SA, Williamson JB, Doty L 2014 Enhanced somatosensory feedback reduces prefrontal cortical activity during walking in older adults. J. Gerontol. A Biol. Sci. Med. Sci. 69, 1422–1428. (doi:10.1093/gerona/glu125) 25112494 10.1093/gerona/glu125 PMC4229993

[48] Frigon A, Gossard J-P 2009 Asymmetric control of cycle period by the spinal locomotor rhythm generator in the adult cat. J. Physiol. 587, 4617–4628. (doi:10.1113/jphysiol.2009.176669) 19675066 10.1113/jphysiol.2009.176669 PMC2768017

[49] Dietz V 2002 Proprioception and locomotor disorders. Nat. Rev. Neurosci. 3, 781–790. (doi:10.1038/nrn939) 12360322 10.1038/nrn939

[50] Palmer E, Ashby P 1992 Corticospinal projections to upper limb motoneurones in humans. J. Physiol. 448, 397–412. (doi:10.1113/jphysiol.1992.sp019048) 1593472 10.1113/jphysiol.1992.sp019048 PMC1176206

[51] Brouwer B, Ashby P 1992 Corticospinal projections to lower limb motoneurons in man. Exp. Brain Res. 89, 649–654. (doi:10.1007/BF00229889) 1644127 10.1007/BF00229889

[52] Kuhtz-Buschbeck JP, Frendel A, Jing B 2014 Arm swing during human gait studied by EMG of upper limb muscles. In Applications, challenges, and advancements in electromyography signal processing (ed. Naik GR), pp. 129–160. IGI Global.

[53] Montague D, Weickert CS, Tomaskovic-Crook E, Rothmond DA, Kleinman JE, Rubinow DR 2008 Oestrogen receptor α localisation in the prefrontal cortex of three mammalian species. J. Neuroendocrinol. 20, 893–903. (doi:10.1111/j.1365-2826.2008.01743.x) 18445128 10.1111/j.1365-2826.2008.01743.x PMC2719673

[54] Hao J et al. 2006 Estrogen alters spine number and morphology in prefrontal cortex of aged female rhesus monkeys. J. Neurosci. 26, 2571–2578. (doi:10.1523/JNEUROSCI.3440-05.2006) 16510735 10.1523/JNEUROSCI.3440-05.2006 PMC6793646

[55] Hjelmervik H, Westerhausen R, Osnes B, Endresen CB, Hugdahl K, Hausmann M, Specht K 2012 Language lateralization and cognitive control across the menstrual cycle assessed with a dichotic-listening paradigm. Psychoneuroendocrinology 37, 1866–1875. (doi:10.1016/j.psyneuen.2012.03.021) 22534404 10.1016/j.psyneuen.2012.03.021

[56] Keenan PA, Ezzat WH, Ginsburg K, Moore GJ 2001 Prefrontal cortex as the site of estrogen's effect on cognition. Psychoneuroendocrinology 26, 577–590. (doi:10.1016/S0306-4530(01)00013-0) 11403979 10.1016/s0306-4530(01)00013-0

[57] Krug R, Born J, Rasch B 2006 A 3-day estrogen treatment improves prefrontal cortex-dependent cognitive function in postmenopausal women. Psychoneuroendocrinology 31, 965–975. (doi:10.1016/j.psyneuen.2006.05.007) 16831520 10.1016/j.psyneuen.2006.05.007

[58] Egner T 2009 Prefrontal cortex and cognitive control: motivating functional hierarchies. Nat. Neurosci. 12, 821–822. (doi:10.1038/nn0709-821) 19554047 10.1038/nn0709-821

[59] Gwin JT, Gramann K, Makeig S, Ferris DP 2011 Electrocortical activity is coupled to gait cycle phase during treadmill walking. Neuroimage 54, 1289–1296. (doi:10.1016/j.neuroimage.2010.08.066) 20832484 10.1016/j.neuroimage.2010.08.066

[60] Heekeren HR, Marrett S, Bandettini PA, Ungerleider LG 2004 A general mechanism for perceptual decision-making in the human brain. Nature 431, 859–862. (doi:10.1038/nature02966) 15483614 10.1038/nature02966

[61] Burhan AM, Subramanian P, Pallaveshi L, Barnes B, Montero-Odasso M 2015 Modulation of the left prefrontal cortex with high frequency repetitive transcranial magnetic stimulation facilitates gait in multiple sclerosis. Case Rep. Neurol. Med. 2015, 251829 (doi:10.1155/2015/251829) 26421201 10.1155/2015/251829 PMC4572429

[62] Juvin L, Le Gal J-P, Simmers J, Morin D 2012 Cervicolumbar coordination in mammalian quadrupedal locomotion: role of spinal thoracic circuitry and limb sensory inputs. J. Neurosci. 32, 953–965. (doi:10.1523/JNEUROSCI.4640-11.2012) 22262893 10.1523/JNEUROSCI.4640-11.2012 PMC6621141

[63] Nathan PW, Smith M, Deacon P 1996 Vestibulospinal, reticulospinal and descending propriospinal nerve fibres in man. Brain 119, 1809–1833. (doi:10.1093/brain/119.6.1809) 9009990 10.1093/brain/119.6.1809

[64] Sejdić E, Fu Y, Pak A, Fairley JA, Chau T 2012 The effects of rhythmic sensory cues on the temporal dynamics of human gait. PLoS ONE 7, e43104 (doi:10.1371/journal.pone.0043104) 22927946 10.1371/journal.pone.0043104 PMC3424126

[65] Kaipust JP, McGrath D, Mukherjee M, Stergiou N 2013 Gait variability is altered in older adults when listening to auditory stimuli with differing temporal structures. Ann. Biomed. Eng. 41, 1595–1603. (doi:10.1007/s10439-012-0654-9) 22956164 10.1007/s10439-012-0654-9

[66] Zifchock RA, Davis I, Higginson J, Royer T 2008 The symmetry angle: a novel, robust method of quantifying asymmetry. Gait Posture 27, 622–627. (doi:10.1016/j.gaitpost.2007.08.006) 17913499 10.1016/j.gaitpost.2007.08.006

[67] Błazkiewicz M, Wiszomirska I, Wit A 2014 Comparison of four methods of calculating the symmetry of spatial-temporal parameters of gait. Acta Bioeng. Biomech. 16, 29–35. (doi:10.5277/abb140104) 24708092

[68] Araújo R, Ferreira JJ, Antonini A, Bloem BR 2015 ‘Gunslinger's gait’: a new cause of unilaterally reduced arm swing. Br. Med. J. 351, 1–5. (doi:10.1136/bmj.h6141) 10.1136/bmj.h6141 PMC4678175 26666758

[69] Harvey M, Milner AD, Roberts RC 1995 An investigation of hemispatial neglect using the landmark task. Brain Cogn. 27, 59–78. (doi:10.1006/brcg.1995.1004) 7748546 10.1006/brcg.1995.1004

[70] Ciçek M, Deouell LY, Knight RT 2009 Brain activity during landmark and line bisection tasks. Front. Hum. Neurosci. 3, 7 (doi:10.3389/neuro.09.007.2009) 19521543 10.3389/neuro.09.007.2009 PMC2694675

[71] Killeen T, Easthope C, Filli L, Lőrincz L, Schrafl-Altermatt M, Brugger P, Linnebank M, Curt A, Zörner B, Bolliger M 2016 Data from: Increasing cognitive load attenuates right arm swing in healthy human walking. Dryad Digital Repository. (doi:10.5061/dryad.2kd0b) 10.1098/rsos.160993 PMC5319362 28280596

