# Supplementary figures and images for: Increasing cognitive load attenuates right arm swing in healthy human walking
Source: R Soc Open Sci. 2017 Jan 25;4(1):160993. doi: 10.1098/rsos.160993 (PMC5319362; doi:10.1098/rsos.160993)

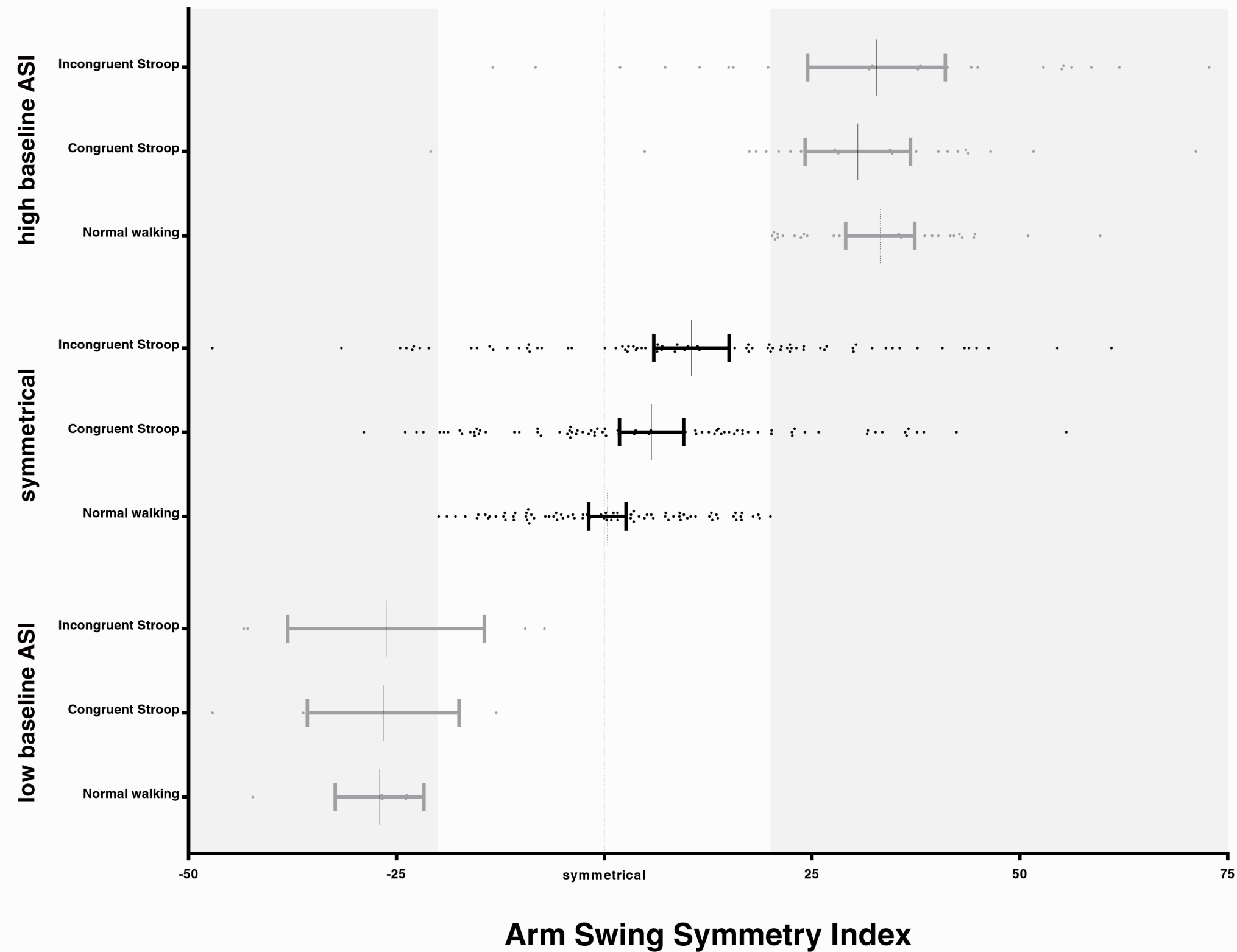

Supplement: Supplementary Figure 1 [file rsos160993supp1.pdf]

# Arm Swing Symmetry Index

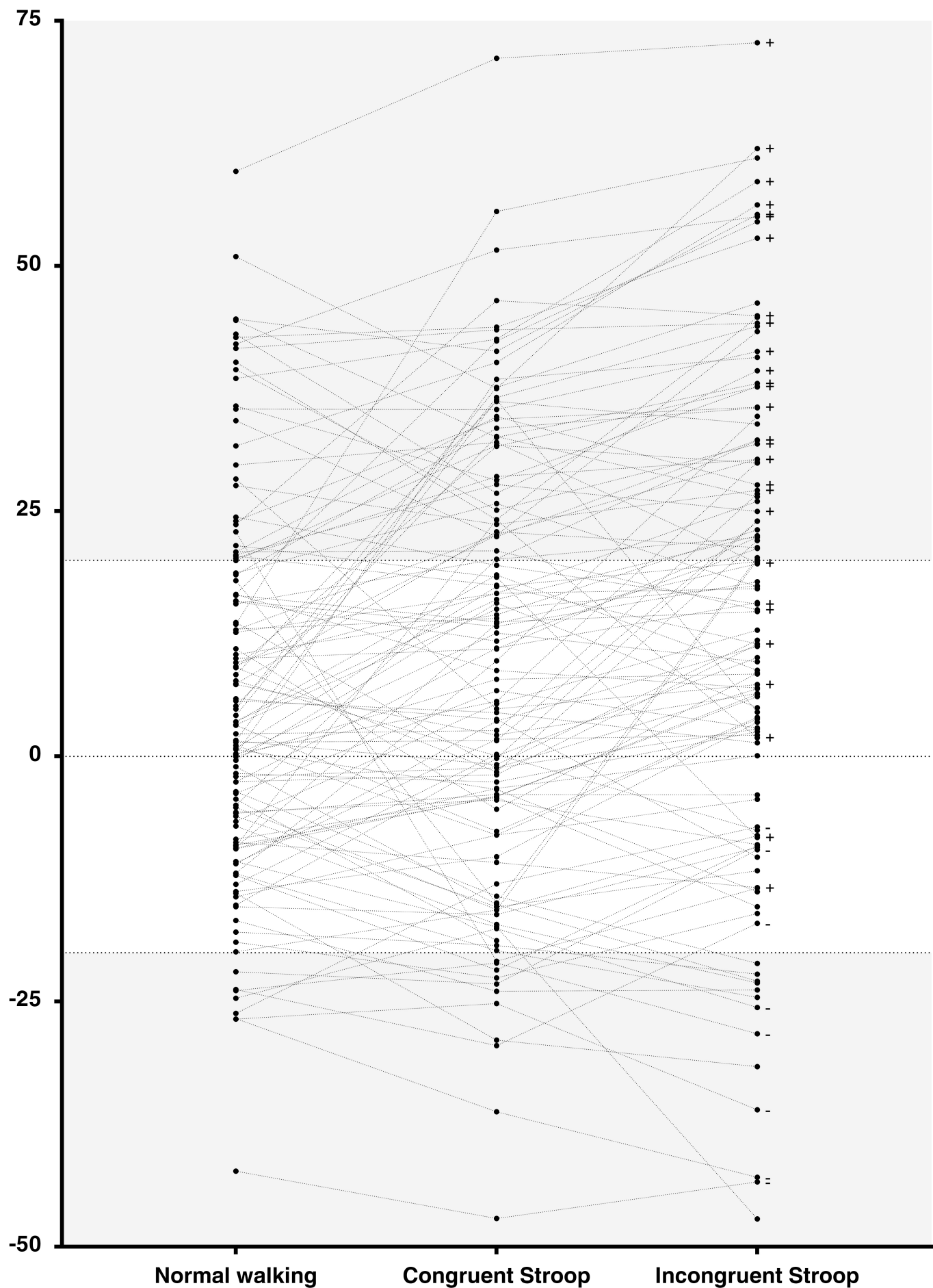

Supplement: Supplementary Figure 2 [file rsos160993supp2.pdf]

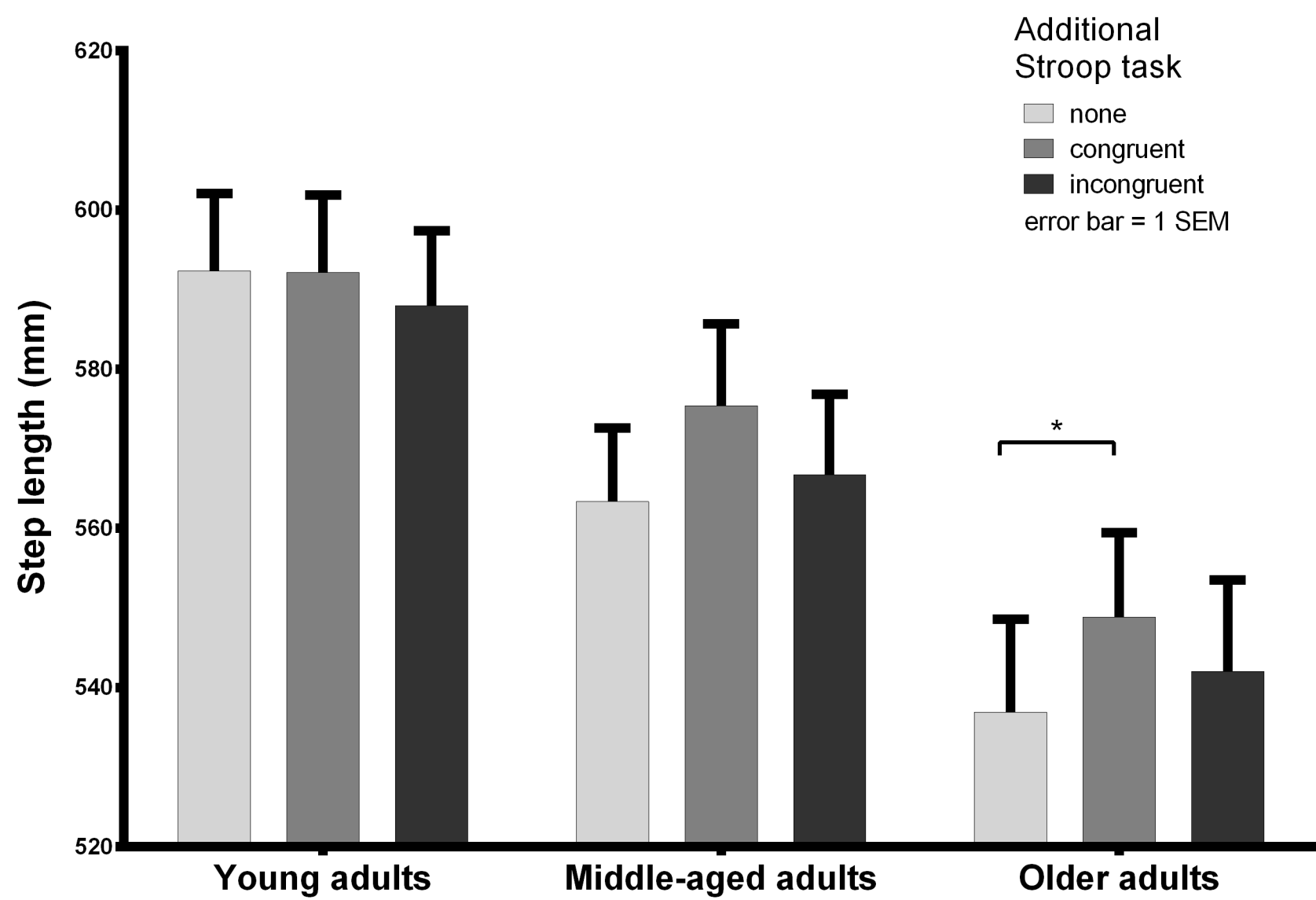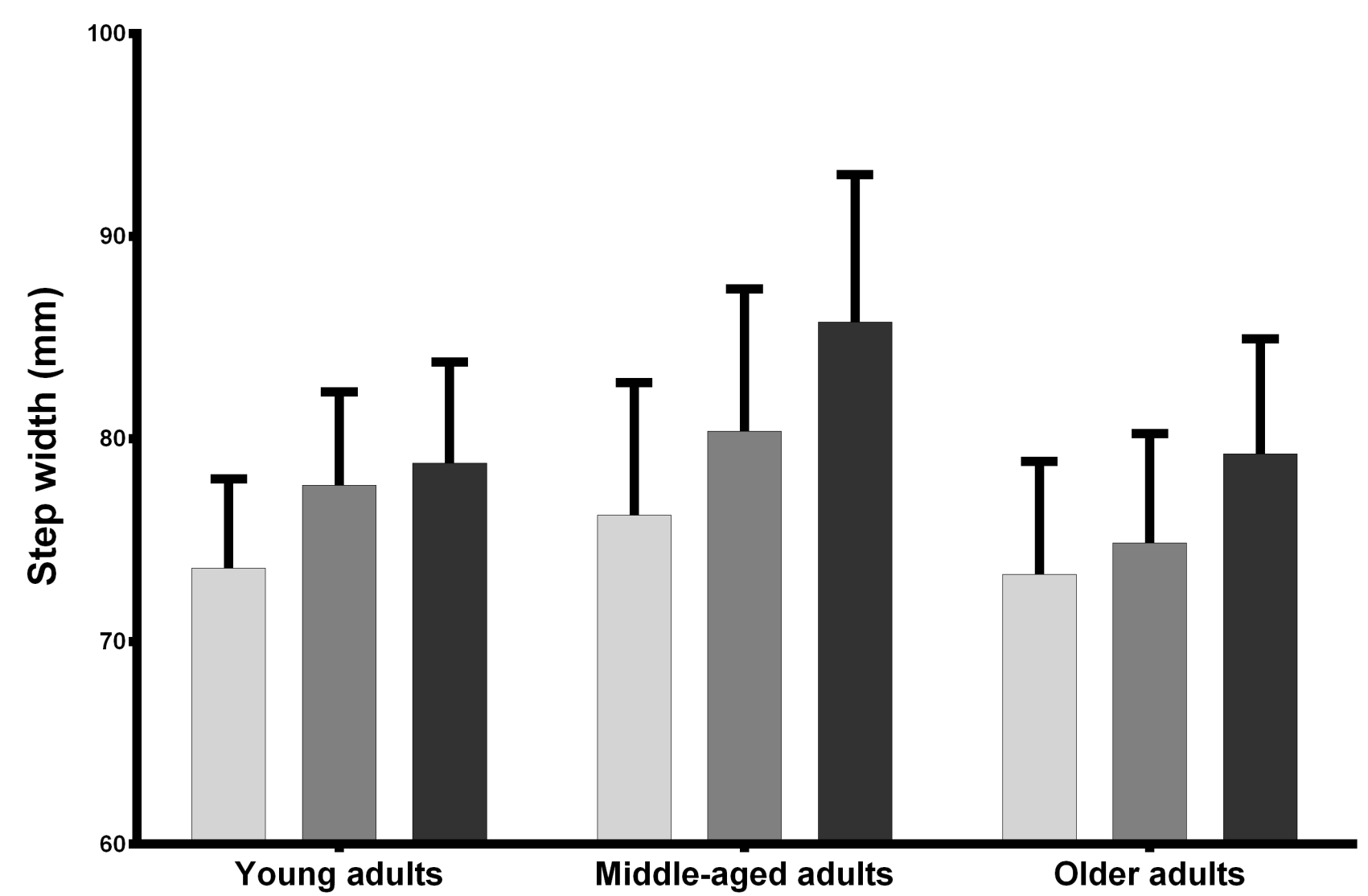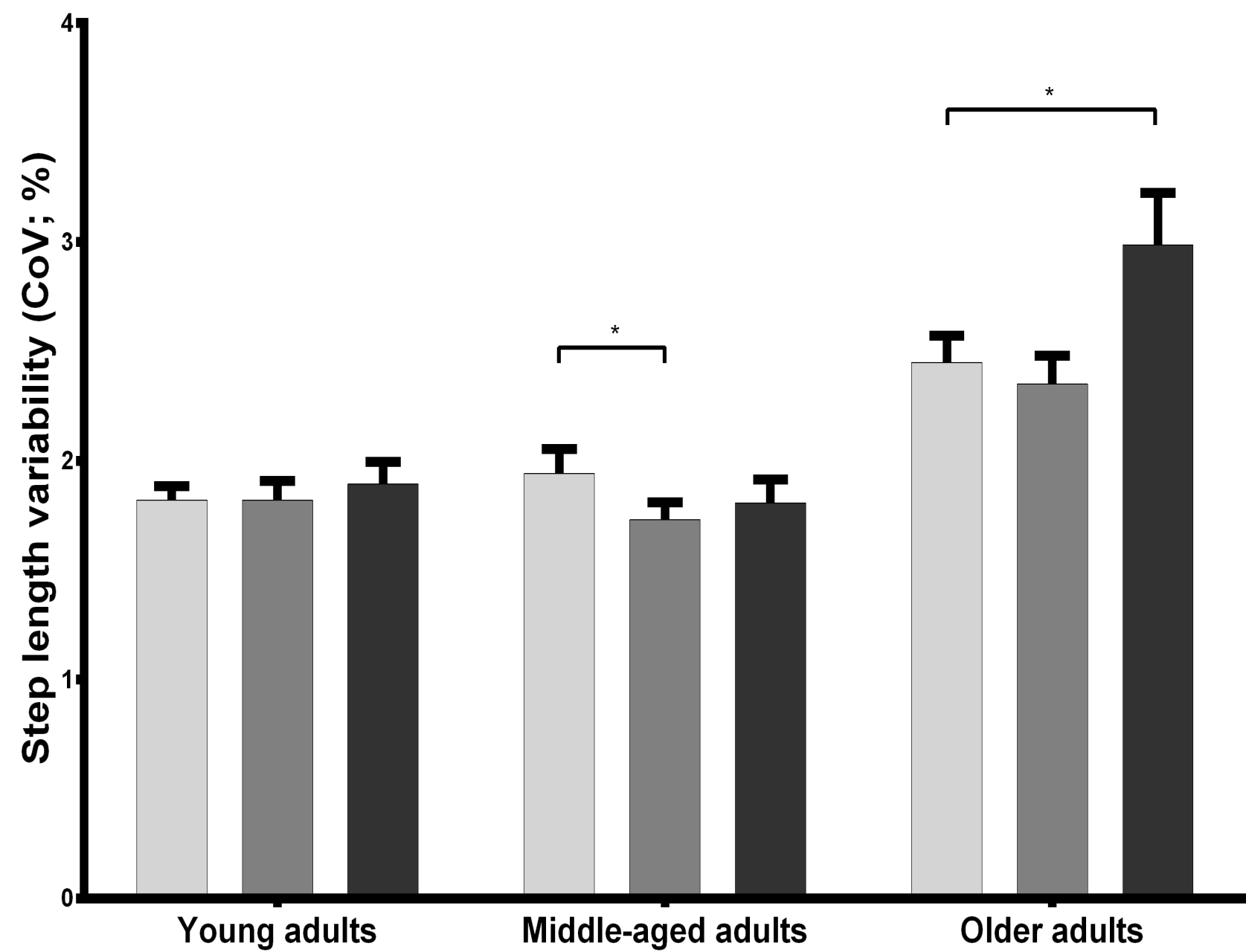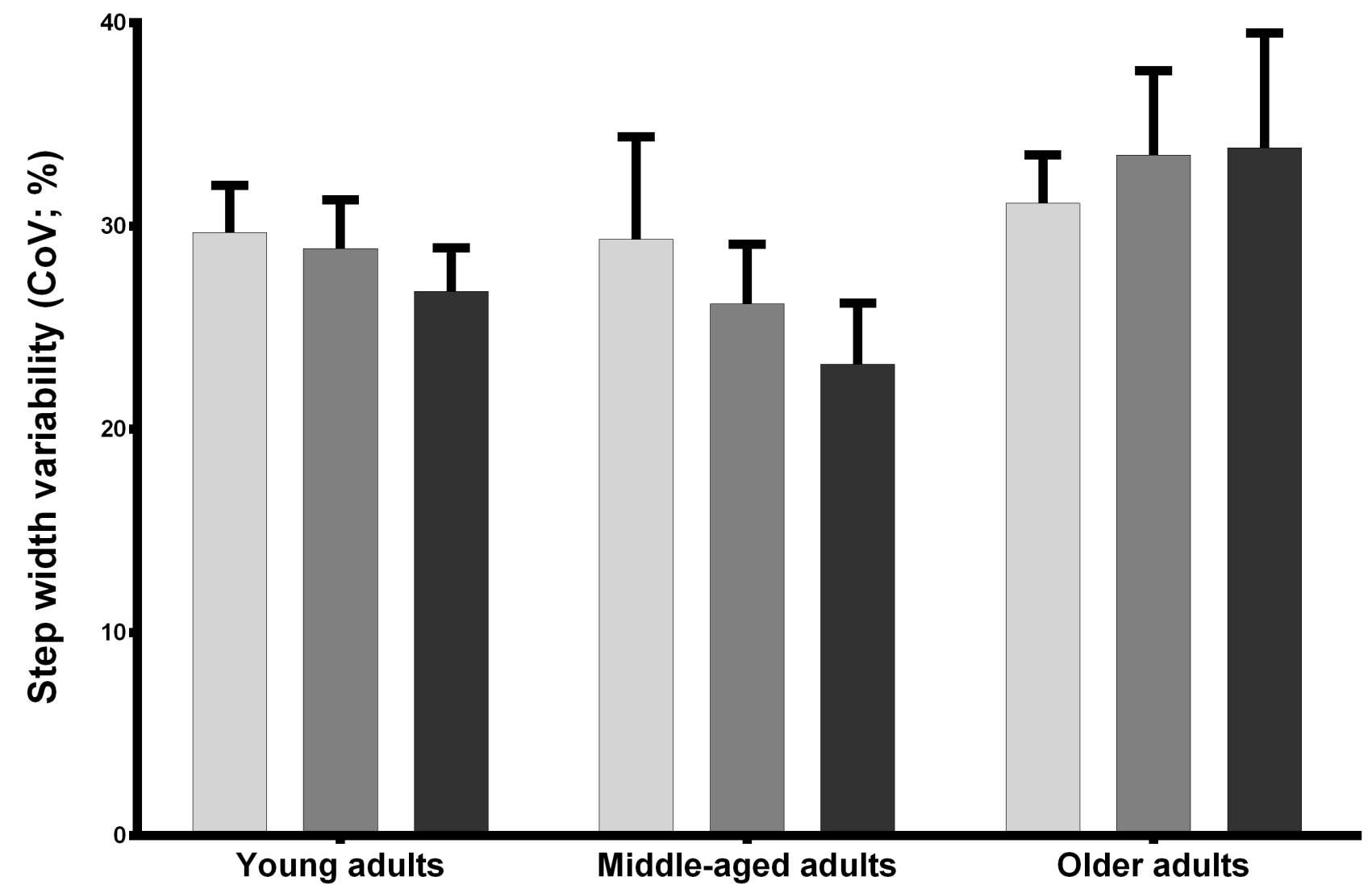

Supplement: Supplementary Figure 3 [file rsos160993supp3.pdf]

# Foot clearance

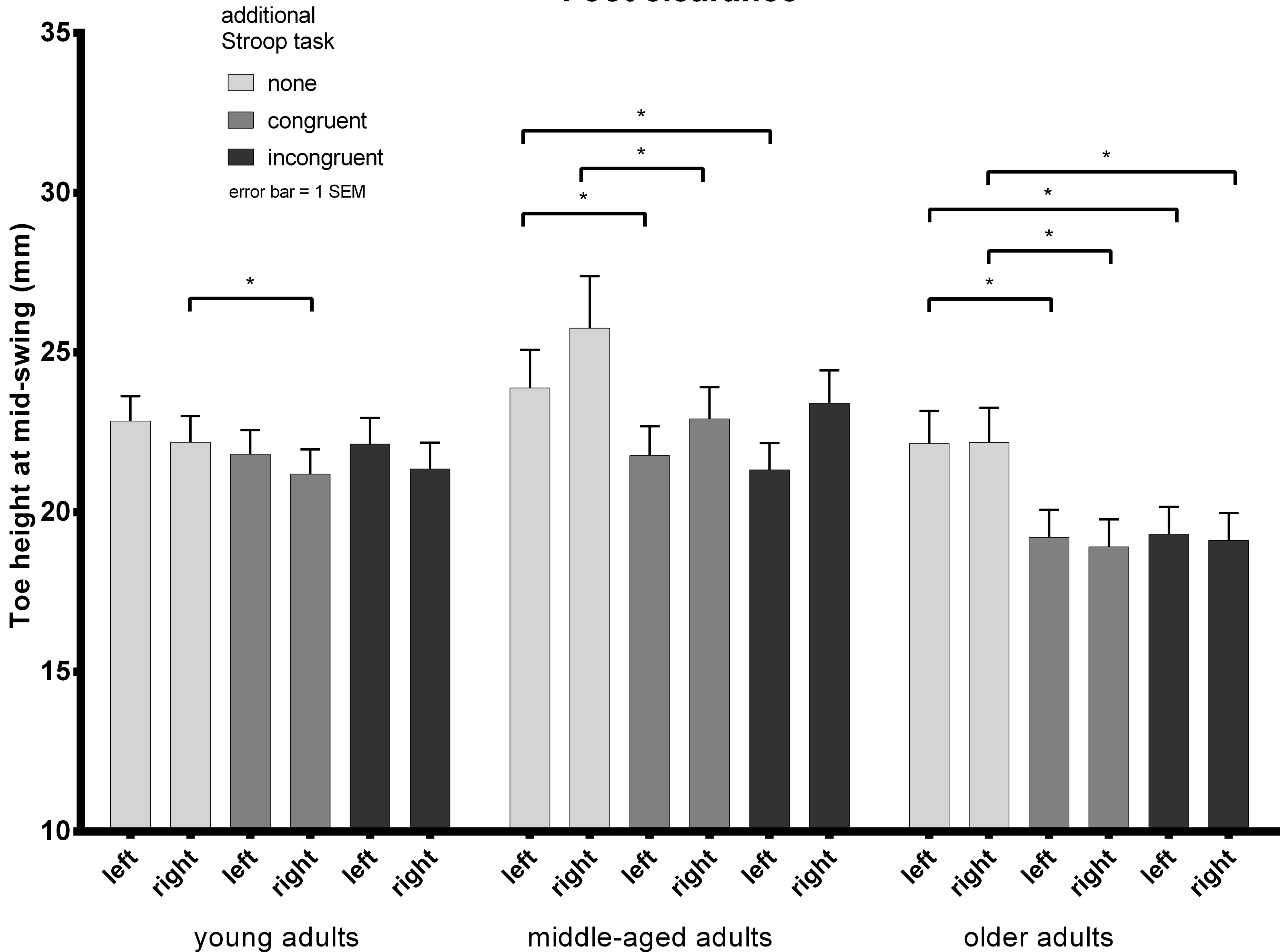

Supplement: Supplementary Figure 4 [file rsos160993supp4.pdf]
