# Supplementary material for: Increasing cognitive load attenuates right arm swing in healthy human walking
Source: R Soc Open Sci. 2017 Jan 25;4(1):160993. doi: 10.1098/rsos.160993 (PMC5319362; doi:10.1098/rsos.160993)
Supplement: Supplementary Table 1 [file rsos160993supp5.docx]

**Supplementary Table 1. Spatiotemporal gait parameters under increasing cognitive load**

|  |  | Step length | | Step width | | Phase dispersion | | | | | Foot clearance | |
| --- | --- | --- | --- | --- | --- | --- | --- | --- | --- | --- | --- | --- |
|  | Additional Stroop task | Mean (mm) | Variability (CoV; %) | Mean (mm) | Variability (CoV; %) | L arm to R arm (% of gait cycle) | L leg to R leg (% of gait cycle) | L arm to R leg (% of gait cycle) | R arm to L leg (% of gait cycle) | L leg to L leg (% of gait cycle) | Left toe height at midswing (mm) | Right toe height at midswing (mm) |
| Young adults (18 – 39 years) | None (baseline) | 592.3±9.8 | 1.82±0.06 | 73.62±4.41 | 29.68±2.32 | 50.96±0.45 | 49.95±0.12 | 62.63±  0.55 | 63.63±0.50 | 87.31±0.55 | 22.8±0.8 | 22.2±0.8 |
|  | Congruent | 592.1±9.8 | 1.82±0.09 | 77.71±4.62 | 28.90±2.39 | 50.96±0.37 | 50.15±0.12 | 62.58±0.53 | 63.71±0.47 | 87.20±0.57 | 21.8±0.8 | **21.2±0.8*** |
|  | Incongruent | 588.0±9.4 | 1.89±0.10 | 78.82±4.97 | 26.78±2.15 | 51.10±0.36 | 50.12±0.11 | 62.79±0.50 | 64.00±0.44 | 87.09±0.52 | 22.1±0.8 | 21.3±0.8 |
| Middle aged adults (40 – 59 years) | None (baseline) | 563.4±9.2 | 1.94±0.11 | 76.24±6.55 | 29.35±5.05 | 51.58±0.31 | 49.98±0.15 | 63.24±0.53 | 65.76±0.68 | 86.78±0.54 | 23.9±1.2 | 25.8±1.6 |
|  | Congruent | 575.4±10.3 | **1.73±0.08*** | 80.37±7.04 | 26.17±2.94 | 51.41±0.35 | 49.90±0.14 | 63.20±0.57 | 65.02±0.58 | 86.89±0.54 | **21.8±0.9*** | **22.9±1.0*** |
|  | Incongruent | 566.7±10.1 | 1.81±0.11 | 85.76±7.28 | 23.20±3.00 | 51.04±0.41 | 49.97±0.10 | 63.49±0.58 | 64.91±0.59 | 86.42±0.59 | **21.3±0.8*** | 23.4±1.0 |
| Older adults (60 – 80 years) | None (baseline) | 536.9±11.7 | 2.45±0.12 | 73.32±5.59 | 31.11±2.38 | 52.10±0.55 | 50.05±0.12 | 65.47±0.67 | 66.68±0.88 | 84.50±0.65 | 22.1±1.0 | 22.2±1.1 |
|  | Congruent | **548.9±10.6*** | 2.35±0.13 | 74.87±5.39 | 33.49±4.16 | 51.32±0.67 | 50.09±0.15 | 65.64±0.76 | 66.12±0.84 | 84.27±0.76 | **19.2±0.9*** | **18.9±0.9*** |
|  | Incongruent | 542.0±11.5 | **2.99±0.24*†** | 79.26±5.67 | 33.84±5.66 | 50.97±0.68 | 50.12±0.15 | 65.90±0.82 | 66.12±0.81 | 83.59±0.80 | **19.3±0.9*** | **19.1±0.9*** |

Mean ± SEM of gait parameters under three walking conditions on a treadmill; normal walking (no additional cognitive load), walking while performing a congruent Stroop task and walking while performing an incongruent Stroop task. Step length is the distance in the progression axis from heel strike to ipsilateral heel strike. Step width is the distance in the lateral axis from heel strike to contralateral heel strike. Phase dispersion is calculated as the point in the time normalised gait cycle at which the coordinated event occurs during the cycle of the second limb expressed as a percentage of the latter, with maximal arm protraction taken as the index event for arm swing and toe-off taken as that for stepping. Foot clearance is reported as the distance in the vertical axis of the toe marker from the treadmill at the point of ipsilateral mid swing. * indicates significant change to baseline walking condition, † indicates significant change between incongruent and congruent Stroop task condition (linear mixed model, p≤0.05). CoV; coefficient of variation.
